# Comparison of therapies of white spot lesions: a systematic review and network meta-analysis

**DOI:** 10.1186/s12903-023-03076-x

**Published:** 2023-06-01

**Authors:** Zunxuan Xie, Lei Yu, Sining Li, Jianing Li, Yuyan Liu

**Affiliations:** 1 Department of Endodontics, Hospital of Stomatology, Jilin University, Jilin, China; 2 Department of Orthodontics, Hospital of Stomatology, Jilin University, Jilin, China; 3 Department of Prosthodontics, Hospital of Stomatology, Jilin University, Jilin, China

**Keywords:** White spot lesions, Remineralization, Network Meta-analysis, Fluoride varnish, CPP-ACP, Resin infiltration

## Abstract

**Objective:**

White spot lesions (WSLs), the earliest evidence of enamel demineralization, are considered amenable to intervention to achieve a remineralized or arrested state of caries. The management of WSLs is quite challenging, and there is no definitive cure as yet. We performed a network meta-analysis to assess the efficacy of seven therapies for WSLs and gave a hierarchy of them.

**Materials and methods:**

We systematically searched the PubMed, EMBASE, Cochrane, and Web of Science databases (last search: July 2022) to identify all relevant studies. We limited our search to studies published in English. Randomized controlled designed in vitro/clinical trials related to the efficacy of the seven therapies for WSLs were included. Data extraction was performed independently by two reviewers. The risk of bias (ROB) 2.0 tool from Cochrane and a previous in vitro methodological tool will be used for the quality assessment. Variations in quantitative light-induced fluorescence (QLF), laser fluorescence (LF), and lesions area were the primary outcome measures. Standard mean difference (SMD) was used as the effect size for the Network meta-analysis (NMA). Consistency and inconsistency tests were conducted. The hierarchy of 7 treatment effects was evaluated using surface probabilities under cumulative ranking (SUCRA). Publication bias was evaluated using a bias plot.

**Results:**

Forty-two articles were included in the systematic review. Thirty-one of them, with a total of 1906 participants, were included in the network meta-analysis. The studies owned a low and moderate risk of bias. This analysis does not suffer from significant inconsistency. The difference between 4 groups ‘self-assembled peptide (SAP) P11-4’, ‘P11-4 + Fluoride Varnish (FV)’, ‘Resin Infiltration (RI)’, ‘casein phosphor peptides-amorphous calcium fluoride phosphate (CPP-ACFP)’ and the 'Control' group was found to be statistically significant. Compared to the ‘FV’ and ‘casein phosphor peptides-amorphous calcium phosphate (CPP-ACP)’ groups, the ‘P11-4 + FV” group and ‘RI” group made a significant difference. The hierarchy was evident in the SUCRA values of 7 therapies. P11-4 + FV and RI were considered effective therapies compared to the control group or the FV group (gold standard group).

**Conclusions:**

The available evidence suggests that resin infiltration and P11-4 in combination with fluoride varnish had advantages over gold standard (FV). The effect of tricalcium phosphate-based drugs and fluoride is not very noticeable. Overall, drugs based on P11-4 and resin infiltration will be better therapies. Using more than two drugs in combination also would increase efficacy.

**Supplementary Information:**

The online version contains supplementary material available at 10.1186/s12903-023-03076-x.

## Introduction

White spot lesions (WSLs), also known as early caries lesions (ECLs), are the earliest evidence of enamel demineralization and remineralization therapy is a trend in treatment [1–3]. WSLs are typically in the international caries detection and assessment system (ICDAS) II 1–2 range [1, 2, 4]. Under physiological conditions, there is a balance between demineralization and remineralization at the enamel surface as a result of altered pH levels [5]. If this balance is disturbed, early caries lesions will appear [3]. It should be mentioned that orthodontic treatments with fixed multibracket appliances hinder the maintenance of oral hygiene, leading to the accumulation of plaque and the progression of dental caries [6–8]. WSLs occur precisely in this way. Moreover, WSLs are assumed to correlate with bracket debonding time, raising concerns about orthodontic WSLs. Orthodontic WSLs are considered active until the time of bracket debonding [6, 9, 10]. The management of caries is undergoing a paradigm shift towards the minimally invasive approach, which emphasizes the prevention, reduction, and reversal of caries in incipient lesions [1, 11, 12]. These early lesions are considered amenable to the intervention to achieve a state of remineralization or arrest of caries. If the process of demineralization is not halted, the intact enamel surface will eventually collapse and cavitate [1, 13–15].

Fluoride-based strategies are the gold standard for preventing and managing WSLs [2, 16, 17]. Fluoride can interact with saliva at the surface and subsurface of the enamel. And then, it can combine with phosphate and calcium ions to form large new crystals containing more fluoride (Fluor-hydroxyapatite), thus improving remineralization [18]. However, current fluoride therapies have been reported to be flawed, especially caries already manifested as white spots [6, 12, 19, 20]. The casein phosphopeptides (CPP) contain multiple phosphoryl sequences that can stabilize calcium phosphate in nano complexes in solutions like amorphous calcium phosphate (ACP). Through their multiple phosphoryl sequences, the CPP binds to ACP in a metastable solution to prevent the dissolution of the calcium and phosphate ions. The casein phosphor peptides- amorphous calcium phosphate (CPP-ACP) also serves as a reservoir for bioavailable calcium and phosphate, thereby promoting remineralization [18, 21]. But compared to fluoride, the mentioned properties of CPP-ACP do not perform well in the treatment results [22–24]. The clinically significant benefit of tricalcium phosphate product over fluoride cannot be performed [6, 25, 26]. The self-assembling peptide P11-4(SAP P11-4) provides a novel opportunity for the remineralization therapy of WSLs through the mechanism of biomimetic mineralization [6, 27–29]. The current findings suggest that P11-4 has superior performance in the treatment of WSLs compared to the gold standard fluoride [12, 15, 30, 31]. Resin infiltration (RI) has also emerged as an effective method to treat WSLs by minimally invasive means [32, 33].

There have been many clinical studies exploring the differences between the methods of treating WSLs, but there isn't a broadly accepted conclusion [32, 34–37]. It is unrealistic to conduct a comparative study of all treatment modalities for WSLs at one time. Traditional meta-analyses have also been performed to compare the differences between two or several treatments [38–41]. In contrast to traditional meta-analyses, network meta-analyses (NMA) allow for the inclusion of evidence from direct and indirect comparisons across different intervention research networks to create multiple hierarchies of intervention effects, even where two interventions comparisons are lacking [42–44]. A comparison of the many treatment options and standard procedures for WSLs is necessary [45]. To date, however, no comparison of WSLs’ therapies has been performed using a network meta-analysis with relatively sufficient evidence. Therefore, this study aimed to perform a systematic review and network meta-analysis to compare the aforementioned therapies for contributing to the establishment of clinical treatment guidelines for WSLs [41].

## Methods and analysis

### Registration

The systematic review and network meta-analysis are reported following the Preferred Reporting Items for Systematic Reviews and Meta-Analyses (PRISMA) statement [46]. The study protocol was registered (registration number: CRD42022343703) with the International Prospective Register of Systematic Reviews (PROSPERO).

### Search strategy

Two researchers (Xie and Yu) independently searched for meta-analysis articles published in the following databases: Web of Science, EMBASE, PubMed, and Cochrane Central Register of Controlled Trials. They used medical topic headings (MeSH) and free-text terms. The search time frame was from January 2007 to June 2022. The search strategies are based on the PICOS principle, which can be found in Supplementary Table.

### Selection of researches and eligibility criteria

The two reviewers (Xie and Yu, blinded to each other) independently completed the screening of the studies using a specifically designed data extraction form. The disagreement will be solved by Li using an inner decision system. Trials were considered eligible according to inclusion and exclusion criteria. The following are detailed criteria in Table 1.

**Table 1:** Inclusion and exclusion criteria

Principle	Inclusion Criteria	Exclusion Criteria
Participant	Participants with WSLs (including post-orthodontic WSLs and non-post-orthodontic WSLs), ECLs, active caries lesions (ACLs) without symptoms, and other superficial demineralization lesions; Artificial lesions with defined in vitro/in vivo demineralization procedures. The lesions mentioned in the text need to meet the definition of WSLs in ICDAS II	Deep caries, root caries, dental fluorosis, as well as other types of dental defects. Participants were found to have congenital or systemic conditions
Interaction & Control	Various therapies for WSLs and ECLs: Resin infiltration; Fluoride varnish; Self-assembling peptide P11-4 with/without fluoride varnish; CPP-ACP; CPP-ACFP; placebo or various control measures. Fluoridated kinds of toothpaste are identified as standard oral health guidelines and are not considered to be specific interventions	Composite resin filler therapy; Fluoride concentration and frequency related therapy; and specific therapies that are either not widely used in the medical field or are only used by individual academics
Outcome	Changes in the values of lesions such as QLF (quantitative light induced fluorescence) and LF (DIAGNOdent measuring pen) or changes in the area of lesion were measured by image analysis, as well as any measurements that indicates the extent of the lesion	Outcome indicators from count data such as progression or completion of caries. Outcome of the color change or visual evaluation of the lesion area like visual analog scale (VAS)
Study design	RCT designed; Completed and published	Retrospective clinical studies, cohort studies and case–control studies, case reports, or reviews
Additional Criteria		Trials were excluded from the analysis if they had no at least 2-week follow-up. Trials were excluded from the analysis if they had no data available for analysis. Trials were excluded from the review if they were found to be plagiarized

The criteria were developed according to the PICOS principles. The selection of the literature requires all content to be satisfied simultaneously. *WSLs* white spot lesions, *ECLs* early caries lesions, *ACLs* active caries lesions, *ICDAS II* international caries detection and assessment system II, *P11-4* self-assembling peptide 11–4, *CPP-ACP* casein phosphor peptides- amorphous calcium phosphate, *CPP-ACFP* casein phosphor peptides-amorphous calcium fluoride phosphate, *QLF* quantitative light-induced fluorescence, *LF* laser fluorescence, *RCT* randomized controlled trials; VAS: visual analog scale

In the research, it was necessary to exclude diseases with similar treatment modalities to WSLs, such as deep caries, root caries, and fluorosis [47–51]. Systemic and structural barriers also limit dental health for individuals with special healthcare needs (SHCN) [52]. For intervention, in contrast to resin infiltration, conventional composite resin filling is contrary to the current treatment philosophy of managing WSLs [53, 54]. We also had to confront several studies that explored drug concentrations, frequency of use, and use of novel forms of treatment [55–57]. We had difficulty performing a network meta-analysis of these unique forms of intervention. For this research, we tend to analyze measures that have specific values. Visual indicators such as visual analog scale (VAS) may introduce a potential bias, which also questions the accuracy of optical indicators [50, 58]. Conventional fluoride varnish has to be applied repeatedly ranging from once every 2 weeks to four topical applications a year to maintain its effectiveness [31, 59]. It is necessary to set a follow-up time ADDIN EN.CITE. Non-RCT designed and plagiarized articles are not eligible for review.

### Data extraction

The following data will be extracted by two blinded reviewers using EXCEL software, Author and journal; Publication year; Study design; Participants and groups; Baseline characters; Intervention; Comparison; Outcome; Results, and Follow-up period. The data will be extracted from the full text or if missing data is present, the author will be contacted via email. The disagreement will be solved by Li using an inner decision system.

### Risk of bias in individual studies

For clinical research, the ROB 2.0 tool from Cochrane will be used for the quality assessment [60]. The risk of bias will be assessed based on the following five parts: randomization process, deviations from intended interventions, missing outcome data, measurement, and selection of the reported results. The overall risk of bias was expressed as 'low risk of bias' if all domains were categorized as low risk, 'some concerns' if a certain concern was raised in at least one area but was not classified as high risk in any other area, or ‘high risk of bias’ if at least one domain has been classified as high risk, or if it has multiple domains with certain concerns [60]. The methodological quality assessment tool for included in vitro study was from previous systematic reviews of in vitro studies [61, 62]. The risk of bias in each article was evaluated according to the description of the following parameters: specimen randomization; single-operator protocol implementation; blinding of the testing machine operator; the presence of a control group; standardization of the sample preparation; outcome mode evaluation; use of all materials according to the manufacturer’s instructions; description of the sample size calculation. If the reviewers stated the parameter, the study received a “YES” for that specific parameter. In the case of missing data, the parameter received a “NO.” The risk of bias was classified regarding the sum of “YES” answers received: 1 to 3 indicated a high bias, 4 to 6 medium, and 7 to 8 indicated a low risk of bias. All quality assessment processes are carried out by two blinded researchers (Xie and Yu), with Li responsible for resolving disputes arising from this process.

### Data analysis

We performed a network meta-analysis to analyze direct and indirect comparisons of the six different therapies and the control treatment using a multivariable meta-analysis model with the STATA 15.1 statistical software (Stata Corp. College Station, Texas, USA).

The outcome of interest is the variation (from baseline to endpoint) in the absolute value of the lesion metric, such as QLF (quantitative photo-induced fluorescence), LF (DIAGNOdent measurement pen), or lesion area, which is typically measured by image analysis. Where studies did not provide a standard deviation (SD) of the change in outcomes, these values were estimated using a correlation coefficient (r) of 0.5 and the following equation:

$$SD_{change}=\surd [SD^{2}_{baseline}+SD^{2}_{final}-(2r\times SD_{baseline}\times SD_{final})]$$

According to the Cochrane Handbook guideline [63]. Since these changes were continuous outcomes by various measurements, the effect sizes were calculated as SMDs and 95% confidence intervals (CIs). The difference between the drugs was considered significant when the 95%CI for SMD did not include 0 (equivalent to *P* < 0.05). We conducted an inconsistency analysis to explore differences between the direct and various indirect effect estimates for the same comparison [42, 64]. Inconsistency between direct and indirect comparisons may indicate transitivity that is not immediately obvious [42, 65]. The side-split test was used to analyze the local inconsistency. After that, a consistency model was used for network meta-analysis. To rank the effects of the treatment regimens, we used surface probabilities under cumulative ranking (SUCRA) [66]. A SUCRA of x% indicates that the intervention achieves x% of the effectiveness of the imaginary intervention; thus, larger SUCRAs indicate more preferable interventions [42]. The forest plot was based on the consistency model. Additionally, publication bias was assessed using a comparison-adjusted funnel plot.

## Results

### Search results

Our search strategy identified 3032 studies from four primary databases. Furthermore, we identified ten additional studies after reviewing the reference lists of all eligible articles and recent systematic reviews. Following the removal of 660 duplicate records removed, 2382 records were evaluated. When 2033 non-RCT records were removed, 349 articles were included in the final eligible assessment. Subsequently, 42 studies fulfilled the requirements of the systematic review [1, 2, 6, 12, 15, 22, 26, 30–32, 34, 36, 37, 67–95]. Eleven studies were excluded from the NMA because the format of the results or the quantity of interventions is not appropriate for use in NMA [22, 26, 34, 67, 71–73, 76, 81, 83, 86]. Among the 31 studies included in NMA, the following treatment conditions were evaluated: CPP-ACP [10]; CPP-ACFP (6 studies); Control (22 studies); FV (21 studies); P11-4(7 studies); P11-4 + FV (5 studies); RI (5 studies). As 2 studies provided 2 additional outcomes, 33 results from 31 studies were included in the meta-analysis.

**Fig. 1:**
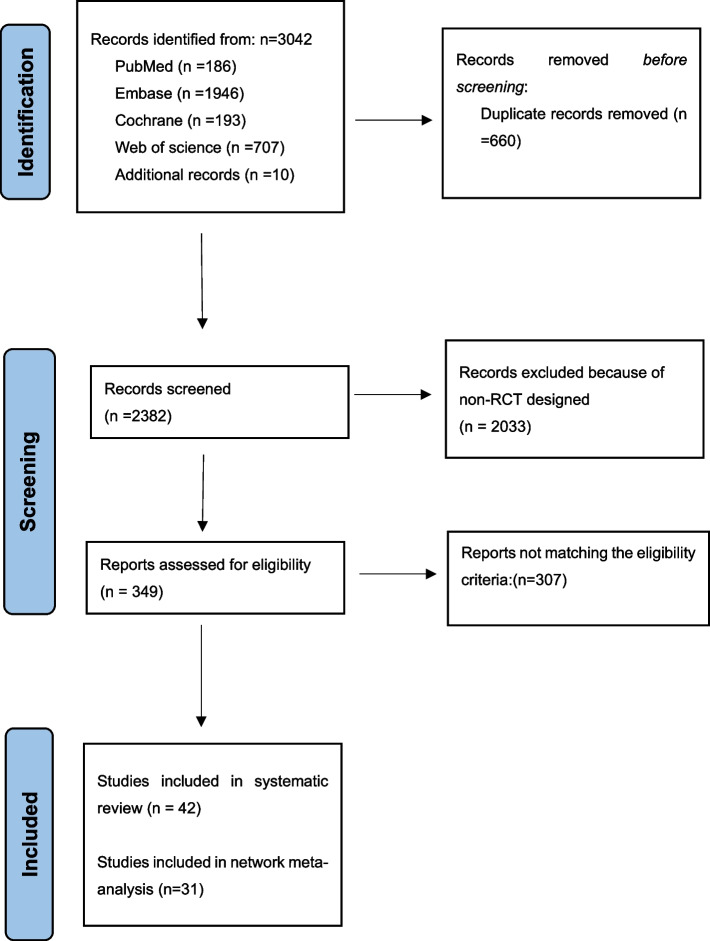
Flowchart diagram of randomized controlled trials of WSL/ECL’s therapies

The flow chart of the literature retrieval process is shown in Fig. 1.

### Characteristics of the included studies

Data extraction results were displayed in the following Table 2. All articles were RCT-designed research. Articles reporting sex ratios were relatively balanced. For most articles, the participants were in the range of children and adolescents. 29/36 in vivo articles were focused on permanent teeth, while 7/36 in vivo articles focused on primary teeth. Six studies did not provide a detailed message regarding the age of the participants because these were in vitro-engineered lesions. Five articles focused on the different kinds of toothpaste used in daily life, which made them lacking the appropriate interventions and absent in NMA [2, 71, 73, 76, 81]. 2 articles provide additional records for NMA [12, 89]. In 42 studies, 35.7% orthodontics WSLs, 4.8% non-orthodontics WSLs, 16.7% WSLs without special introductions, 7.1% ACLs,19.0% ECLs, 2.4% molar incisor hypo-mineralization (MIH) [75] and 14.3% artificial lesions compose all lesions. But a single ACL study with a description of ICDAS = 2 resulted in its inclusion in NMA [68]. Due to the uncertainty of using fluoride toothpaste in oral education (Some articles have explicit descriptions, others do not), we did not consider the efficacy of fluoride toothpaste in this study. Two studies were on occlusal surface lesions [15, 30], where the outcomes were generally consistent with those of smooth surface lesions. One study studied both occlusal surface and smooth surface [95] with a mild difference observed between them, while the rest of the studies were on smooth surface lesions or smooth surface lesions associated with orthodontic brackets.

**Table 2:** Characteristics of systematic reviews’ study

Study	Participants(M/F)	Age	Teeth	Lesion type		Interventions	Follow-up	NMA	MEAS		Outcomes
Giray 2018	23(13/10)	10.78 ± 2.08	81	WSLs	RI	12(45)	6 M	Y	DIAGNOdent	RI	-7 ± 3.67
					FV	11(36)				FV	-2.36 ± 37
Vollú 2019	67(41/26)	3.62 ± 1.07	117	ACL	SDF	34(65) → 31(61)	12 M	N	ICDAS	SDF	Arr 55 Act 7
					ART	33(52) → 26(45)				ART	Arr 43 Act 2
Souza 2021	60(36/24)	6·8	NI	ACL (ICADAS = 2)	TiF4	20 → 16	18 M	Y	QLF	TiF4	-17.5 ± 3.9 → -14.6 ± 4.0
					FV	20 → 16				FV	-15.7 ± 3.2 → -14.9 ± 2.2
					Control	20 → 16				Con	-16.4 ± 3.2 → -14.4 ± 2.0
Jablonski 2020	108	NI	108	Artificial WSLs	P11-4 + FV	36	1 M	Y	QLF	P11-4 + FV	-9.8 ± 3.1 → -5.3 ± 2.79
					FV	36				FV	-10.12 ± 3.13 → -8.29 ± 2.07
					Control	36				Con	-9.7 ± 2.05 → -9.53 ± 2.51
Gözetici 2019	21(10/11) *4	15.4 ± 2.5	NI	WSLs	RI	21 → 20	6 M	Y	DIAGNOdent	RI	-23.25 ± 18.21
					P11-4	21 → 20				P11-4	-8.15 ± 13.89
					FV	21 → 20				FV	-10.1 ± 10.31
					Control	21 → 20				Con	-4.15 ± 9.72
Karabekiroğlu 2017	41	14–20	178	orthodontics WSLs	CPP-ACP	20 → 16(89)	36 M	Y	DIAGNOdent	CPP-ACP	13.06 ± 5.90 → 4.76 ± 2.48
					Control	21 → 18(89)				Con	12.45 ± 6.52 → 8.20 ± 4.38
Yin, W. 2013	463(237/226)	11.1 ± 0.78	NI	WSLs	Argine + MFP	153 → 144	6 M	N	QLF	Argine + MFP	-9.17 ± 1.96 → -7.95 ± 1.82
					NaF	155 → 147				NaF	-9.24 ± 2.16 → -8.43 ± 2.07
					Control	155 → 147				Con	-9.06 ± 1.82 → -8.48 ± 2.24
Abdellatif 2021	79(32/47)	5.33 ± 1.0	237	ACL	SDF	40(121) → 27(82)	12 M	N	ICDAS	SDF	Arr 81 Act 1
					ART	39(116) → 26(85)				ART	Arr 80 Act 5
Rechmann 2018	37(21/16)	15.9 (13.1–26.0)	579	orthodontics WSLs	CPP-ACFP + CPP-ACP	19(292) → 17(260)	12 M	N	ICDAS	CPP-ACFP + CPP-ACP	21.9 ± 1.3 → 22.3 ± 1.4
					Control	18(287) → 18(287)				Control	21.1 ± 1.3 → 22.5 ± 1.5
Batayneh 2020	114(62/52)	4.5 ± 0.5	NI	ECLs	CPP-ACP + NaF	37(81) → 35(77)	6 M	Y	QLF	CPP-ACP + NaF	2.95 ± 2.3
					NaF	42(75) → 41(71)				NaF	4.08 ± 2.8
					CPP-ACP	35(92) → 31(83)				CPP-ACP	3.69 ± 2.7
Gokce 2017	45	NI	45	Artificial permanent WSLs	Novamin	15	2W	N	QLF	Novamin	6.44 ± 0.29
					NaF	15				NaF	5.41 ± 0.6
					probiotic	15				probiotic	3.26 ± 0.52
Alkilzy 2018	70(42/28)	10 ± 2.7	NI	ECLs	P11-4 + FV	31 → 30	6 M	Y	DIAGNOdent	P11-4 + FV	-18.6 ± 19.8
					FV	34 → 32				FV	-1.1 ± 25.8
Bröchner 2011	60(27/33) → 50	15.2(13–18)	NI	Orthodontic WSLs	CPP-ACP	22	1 M	Y	QLF	CPP-ACP	-6.68 ± 0.58 → -4.45 ± 1.82
					Control	28				Con	-7.04 ± 1.65 → 4.51 ± 2.46
Doberdoli 2020	90(32/58)	11.833 ± 2.377	30	ECL	FV	30 → 23	12 M	Y	DIAGNOdent	FV	5.5 ± 7.8
					P11-4 + FV	30 → 27				P11-4 + FV	-8.5 ± 5.9
					P11-4 + matrix	30 → 27				P11-4 + matrix	-7.7 ± 7.8
Restrepo 2016	51(35/17)	10.25 ± 1.14	51	MIH	FV	26	1 M	Y	QLF	FV	-7.47 ± 0.43 → -6.32 ± 0.5
					Control	25				Con	-7.22 ± 0.40 → -6.43 ± 0.64
Villalpando 2021	123(61/62)	3–6	NI	WSLs	NaF	45	3W	N	DIAGNOdent	NaF	17.1 ± 1.9 → 14.94 ± 2.07
					NaF + HA	39				NaF + HA	17.13 ± 2.05 → 12.77 ± 2.34
					CPP-ACFP	39				CPP-ACFP	17.12 ± 2.22 → 12.32 ± 2.45
Güçlü 2016	21(13/8)	8–15	113	Non orthodontic WSLs	CPP-ACP + FV	6	3 M	Y	DIAGNOdent	CPP-ACP + FV	16.5 ± 2.0 → 3.95 ± 2.6
					FV	5				FV	16.9 ± 2.1 → 6.18 ± 3.0
					CPP-ACP	4				CPP-ACP	16.7 ± 1.6 → 3.16 ± 1.3
					Control	6				Con	16.9 ± 2.2 → 6.42 ± 3.1
Beerens 2018	51(27/24)	15.32 ± 1.6	NI	Orthodontic ECLs	CPP-ACFP	25	12 M	Y	QLF	CPP-ACFP	–8.07 ± 1.39 → –6.25 ± 2.36
					control	26				Con	–8.94 ± 1.72 → –7.10 ± 2.79
Beerens 2010	54(23/31)	15.5 ± 1.6	NI	Orthodontic ECLs	CPP-ACFP	27	3 M	Y	QLF	CPP-ACFP	–8.45 ± 1.17 → –7.52 ± 1.78
					Control	27				Con	–9.10 ± 1.75 → –7.96 ± 2.76
He, T. 2016	211	16.9 (12–25)	528	Orthodontic WSLs	FV	69	6 M	Y	QLF	FV	-13.59 ± 3.75 → -10.91 ± 3.42
					FM	70				FM	-13.15 ± 3.75 → -11.03 ± 3.15
					Control	72				Con	-13.21 ± 3.39 → -12.14 ± 3.02
Srisilapanan 2013	331(182/149)	11.3 ± 0.2	NI	ECLs	Arigine + MFP	166	6 M	N	QLF	Arigine + MFP	-8.56 ± 2.25 → -7.65 ± 1.79
					MFP	165				MFP	-8.68 ± 2.31 → -7.97 ± 2.09
Sitthisettapong 2015	79(38/41)	37.51 ± 2.93 month	NI	ECLs	CPP-ACP	40	12 M	Y	QLF	CPP-ACP	-13.27 ± 3.98 → -12.39 ± 4.26
					Control	39				Con	-13.80 ± 4.30 → -11.97 ± 4.03
Kaaij 2015	32	13.3 (10.0–16.6)	NI	Orthodontic WSLs	FR	11	6W	N	QLF	FR	-11.6 ± 5.0
					Control	21				Con	-10.3 ± 3.0(final value)
Guo, X. 2022	130(76/54)	18.5 ± 3.9	NI	ECLs	FV	65	3 M	Y	QLF	FV	3.86 ± 9.05
					Control	65				Control	0.61 ± 8.27
Singh 2016	41(18/23)	18.31 ± 3.34	NI	Orthodontic WSLs	CPP-ACP	14	6 M	Y	DIAGNOdent	CPP-ACP	119.07 ± 36.27 → 100.64 ± 42.33
					FV	13				FV	105.54 ± 25.20 → 88.85 ± 30.41
					Control	14				Con	131.43 ± 41.42 → 118.71 ± 46.46
Lena 2021	30	NR	30	Artificial bovine WSLs	P11-4	10	3W	Y	QLF	P11-4	14.39 ± 6.94
					FV	10				FV	10.78 ± 11.42
					Control	10				Control	17.66 ± 4.91
Turska 2016	81(47/34)	3.8 ± 1.3	346	ECLs	RI + FV	41	12 M	N	ICDAS	RI + FV	Arr 31 Act 10
					FV	40				FV	Arr 13 Act 27
Yuan 2013	52	NI	NI	Artificial Permanent WSLs	RI	13	1.5 M	N	QLF		Not suitable
					CPP-ACP	13					
					FV	13					
					Control	13					
Bailey 2009	45(22/23)	15.5 (12.3–18.9)	NI	Orthodontic WSLs	CPP-ACP	23(207)	3 M	N	ICDAS	CPP-ACP	pro 10 sta 48 re 149
					Control	22(201)				Control	pro 3 sta 80 re 118
Simon 2022	60(25/35)	13–15	NI	Orthodontic WSLs	RI	27	12 M	Y	Area change	RI	15.56 ± 12.6 → 2.17 ± 2
					CPP-ACP	29				CPP-ACP	11.76 ± 6.8 → 2.6 ± 2.1
Ciftci 2018	39(17/22)	8·16	96	Orthodontic WSLs	RI	21	3 M	Y	DIAGNOdent	RI	11.02 ± 2.63 → 3.22 ± 1.32
					FV	18				FV	12.25 ± 2.73 → 6 ± 2.42
Kannan 2019	12(5/7)	14–30	193	Orthodontic WSLs	RI	6	6 M	Y	DIAGNOdent	RI	4.48 ± 1.42 → 1.48 ± 0.81
					FV	6 → 5				FV	4.60 ± 1.29 → 1.08 ± 0.51
Sedlakova 2020	44(18/26)	27.1 (15–39)	88	Non orthodontic WSLs	P11-4 + FV	40	9 M	Y	DIAGNOdent	P11-4 + FV	6.7 ± 5.3 → 6.8 ± 5.7
					FV	40				FV	6.5 ± 4.9 → 6.4 ± 5.2
Sedlakova 2020	44(18/26)	27.1 (15–39)	88	Non orthodontic WSLs	P11-4	43	3 M	Y	DIAGNOdent	P11-4 + FV	6.7 ± 5.3 → 6.7 ± 4.5
					Control	43				FV	6.5 ± 4.9 → 6.5 ± 4.6
Welk 2020	23(10/13)	15.4	46 → 40	Orthodontic WSLs	P11-4	23 → 20	6 M	Y	Area change	P11-4	-2.7 ± 1.7
					Control	23 → 20				Control	-1.5 ± 1.3
Kobeissi 2020	9(4/5)	11.11 ± 3.8	NI	WSLs	P11-4	20	6 M	Y	DIAGNOdent	P11-4	-41.39 ± 16.73%
					FV	20				FV	-32.72 ± 7.84%
Bröseler2020	37(17/20)	21.8 ± 5.9	90	WSLs	P11-4	36	6 M	Y	Area change	P11-4	1 ± 0.74 → 0.844 ± 0.215
					FV	36				FV	1 ± 0.67 → 1.029 ± 0.235
Bröseler2020	37(17/20)	21.8 ± 5.9	88	WSLs	P11-4 + FV	36	12 M	Y	Area change	P11-4 + FV	1 ± 0.74 → 0.862 ± 0.352
					FV	36				FV	1 ± 0.67 → 1.068 ± 0.401
Üstün 2019	32(16 × 2)	NI	16	Artificial permanent WSLs	P11-4	8	1 M	Y	DIAGNOdent	P11-4	11.0 ± 2.0 → 4.1 ± 0.4
					FV	8				FV	13.8 ± 2.4 → 10.1 ± 2.2
					CPP-ACFP	8				CPP-ACFP	13.5 ± 1.9 → 8.5 ± 2.3
					Control	8				Con	11.6 ± 3.9 → 10.3 ± 3.0
Heravi 2018	24(11/13)	16 ± 3	NI	Orthodontic WSLs	CPP-ACFP	12	3 M	Y	Area change	CPP-ACFP	-3.34 ± 1.08
					Control	12				Con	-0.61 ± 0.58
Tomaževič 2022	42(28/14)	17.4 ± 2.8	NI	Orthodontic WSLs	FV	21	6 m	Y	DIAGNOdent	FV	2.8 ± 1.3 → 2.0 ± 1.9
					Control	21				Con	3.1 ± 2.6 → 2.0 ± 1.7
Memarpour 2015	90	21.20 ± 6.76 Month	NI	WSLs	Control	31	12 M	Y	Area change	Control	-0.1 ± 1.12
					FV	29				FV	-0.51 ± 0.56
					CPP-ACP	30				CPP-ACP	-0.63 ± 0.62
Mehta 2013	45	NI	45	Artificial permanent WSLs	CPP-ACP	15	3W	Y	Light fluorescence device	CPP-ACP	1.47 ± 0.17 → 1.05 ± 0.06
					CPP-ACFP	15				CPP-ACFP	1.47 ± 0.53 → 0.95 ± 0.06
					Control	15				Control	1.55 ± 0.18 → 1.01 ± 0.04
Llena 2015	80	6–14	NI	ECLs	CPP-ACP	20	3 M	Y	DIAGNOdent	CPP-ACP	4.91 ± 3.28 → 3.77 ± 3.33
					CPP-ACFP	20				CPP-ACFP	4.7 ± 3.42 → 3.12 ± 3.11
					FV	20				FV	5.23 ± 4.47 → 4.09 ± 3.60
					Control	20				Control	4.44 ± 3.95 → 3.96 ± 2.31

*NI* no information, *WSLs* white spot lesions, *ECLs* early caries lesions, *ACL* active caries lesions, *MIH* molar incisor hypo-mineralization, *RI* resin infiltration, *FV* fluoride varnish, *FM* fluoride film, *FR* fluoride rinse, *SDF* silver diamine fluoride, *ART* atraumatic restorative treatment, *Arr* arrested, *Act* active, *MFP* sodium monofluorophosphate; In the intervention section, the number in parentheses refers to the number of teeth or the number of lesions

### Results of ROB assessment

**Fig. 2:**
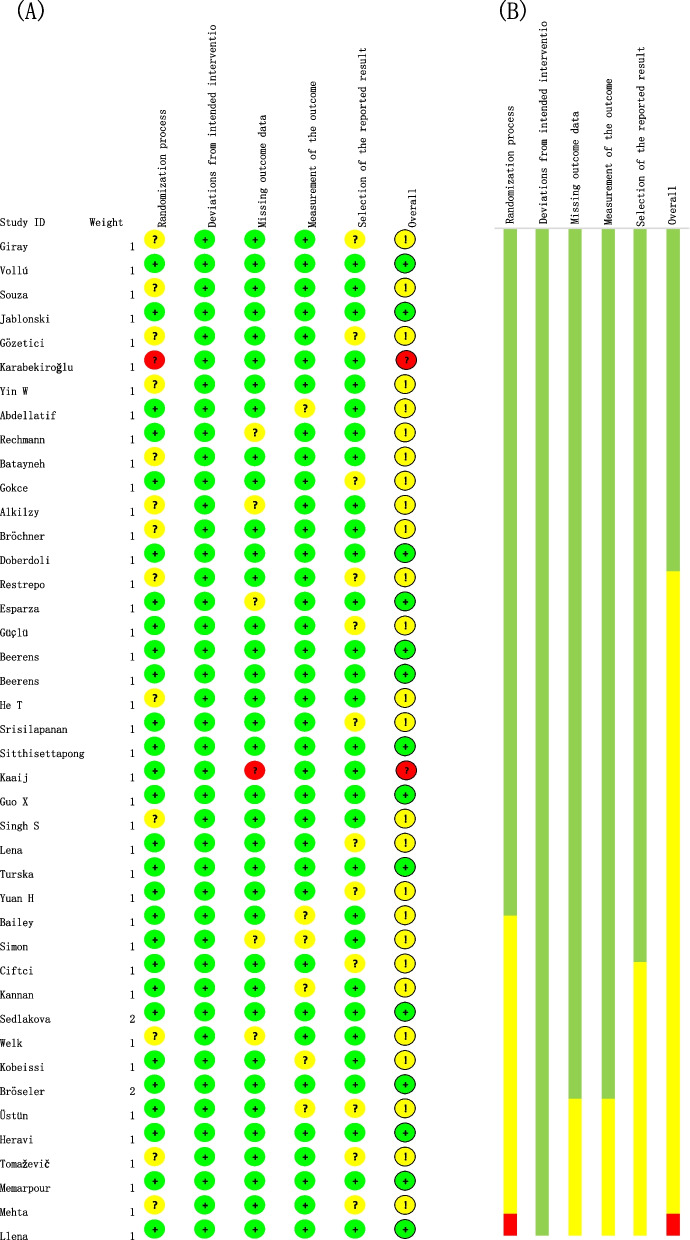
Risk of bias for clinical studies. According to the ROB 2 tool, the risk offset evaluation was carried out from five aspects. Green means low risk, yellow means some concern, and red means high risk. In addition, the overall evaluation results and a bar chart are also shown in the graph

Thirty-six clinical articles were evaluated by ROB 2.0 for the risk of bias. Figure 2 provides details of ROB evaluation in each included clinical study. Overall, 12 articles were judged to be of low ROB, 22 of moderate ROB, and the remaining two were assessed as high ROB. The majority of studies receive a "yellow" rating because there was no information for randomized queue concealment. The other part is that there is no guarantee of the blinded method of the assessor in evaluating the results and whether the procedures were by a pre-specified analysis plan. There are also risks associated with the absence of a specific description of the bias of the outcomes. One of the two high-risk studies was due to the high-risk assessment obtained during the concealment of randomized cohorts, and the other was due to failure to guarantee the impact of loss to follow-up. Table 3 showed the ROB result of the six in vitro studies. Most of the manuscripts involved were counted with a medium or low risk of bias. The sources of risk are from the sample size calculation, single operator, and operator blinded parameters.

**Table 3:** Risk of bias for in vitro study

Study	Specimen Randomization	Single Operator	Operator Blinded	Control Group	Standardized Specimens	Outcome Mode	Manufacturer’s Instructions	Sample Size Calculation	ROB
Jablonski	Yes	Yes	Yes	Yes	Yes	Yes	Yes	Yes	
Gokce	Yes	No	No	Yes	Yes	Yes	No	Yes	
Lena	Yes	No	No	Yes	Yes	Yes	Yes	No	
Yuan	Yes	No	Yes	Yes	Yes	Yes	Yes	No	
Üstün	Yes	No	No	Yes	Yes	Yes	Yes	No	
Mehta	Yes	No	Yes	Yes	Yes	Yes	Yes	Yes	

According to the ROB 2 tool, the risk offset evaluation was carried out from eight aspects. Green means low risk (7–8 scores), yellow means some concern (4–6 scores), and red means high risk (1–3 scores). *ROB *risk of bias

### Network meta-analysis

**Fig. 3:**
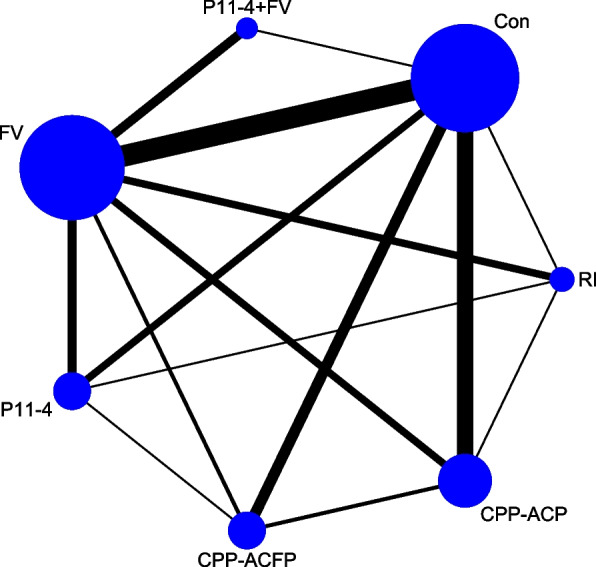
Network Map

This network meta-analysis included a total of 1906 people with 33 outcomes. Figure 3 showed the network map.

#### Network plot

The network of direct treatment comparisons for the changes in absolute values of the outcomes of the WSLs is illustrated in Fig. 3. The sizes of the node reflect the number of matching trials. As shown in the network plot, the ‘FV’ (22 outcomes) and ‘Control’ groups (22 outcomes) were included in the largest number of treatment comparisons, followed by the ‘CPP-ACP’ (10 outcomes) and ‘P11-4’ (7 outcomes), while the ‘CPP-ACFP’ (6 outcomes), ‘RI’ (5 outcomes) and ‘P11 + FV’ (5 outcomes) groups were less. There were 15 direct comparisons. The lines link direct comparisons, and the thickness of the lines represents the number of trials that compare the two therapies. There were 15 pair-to-pair direct comparison groups. The most frequent intercomparison in the included literature was “FV group VS Control group” (12 direct comparisons), followed by the “CPP-ACP group VS Control group” (8 direct comparisons), “CPP-ACFP group VS Control group” (5 direct comparisons), “FV VS P11-4” (5 direct comparisons) and “FV VS P11-4 + FV” (5 direct comparisons). The other specific quantities are also represented in Table 4.

**Table 4:** the results of the side split test

Side	Comparison	Direct		Indirect		Difference		
	Number	Estimate	Std.Err	Estimate	Std.Err	Estimate	Std.Err	P >|z|
A B	2	0.209	0.387	0.280	0.331	-0.706	0.510	0.890
A C	5	0.468	0.240	0.201	0.568	0.267	0.621	0.667
A D	1	0.246	0.413	0.146	0.298	0.100	0.508	0.843
A E	2	-0.644	0.662	-0.016	0.316	-0.628	0.734	0.392
A F^		-	-	-0.54	0.316	-	-	-
A G^		-	-	-0.52	0.327	-	-	-
B C	8	0.087	0.177	0.633	0.402	-0.546	0.437	0.212
B D	3	0.154	0.283	-0.244	0.248	0.399	0.376	0.289
B E^		-	-	-0.38	0.250	-	-	-
B F^		-	-	-0.78	0.281	-	-	-
B G	1	-0.452	0.498	-0.908	0.334	0.457	0.599	0.446
C D	12	-0.324	0.148	0.072	0.301	-0.396	0.335	0.238
C E	4	-0.589	0.279	-0.517	0.327	-0.072	0.430	0.867
C F	1	-1.229	0.491	-0.868	0.286	-0.360	0.568	0.526
C G	1	-1.394	0.536	-0.804	0.299	-0.590	0.613	0.336
D E	5	-0.396	0.249	-0.135	0.353	-0.262	0.430	0.543
D F	5	-0.668	0.222	-1.422	0.889	0.754	0.915	0.410
D G	4	-0.666	0.299	-0.769	0.467	0.102	0.554	0.853
E F^		-	-	-0.4	0.291	-	-	-
E G	1	-1.106	0.521	-0.62	0.356	-1.044	0.630	0.098
F G^		-	-	0.01	0.327	-	-	-

A: CPP-ACFP B:CPP-ACP C: Control D: FV E: P11-4 F: P11-4 + FV G: RI

^: These comparisons had only indirect evidence of comparison

#### Consistency and inconsistency analysis

We performed an inconsistency analysis to identify potential inconsistencies between direct and indirect comparisons. The results indicated that there were no significant differences between the direct comparison and the indirect comparison (χ2 = 9.05, P = 0.9388). We also performed the local inconsistency test; the results of the side split test in Table 4 showed that there was no significant difference between the indirect comparison and direct comparison in 15 groups(P > 0.05). Six comparisons lack the results of direct comparisons but only indirect comparisons, which can be also shown in Fig. 3.

#### Forest plot with the result of NMA

**Fig. 4:**
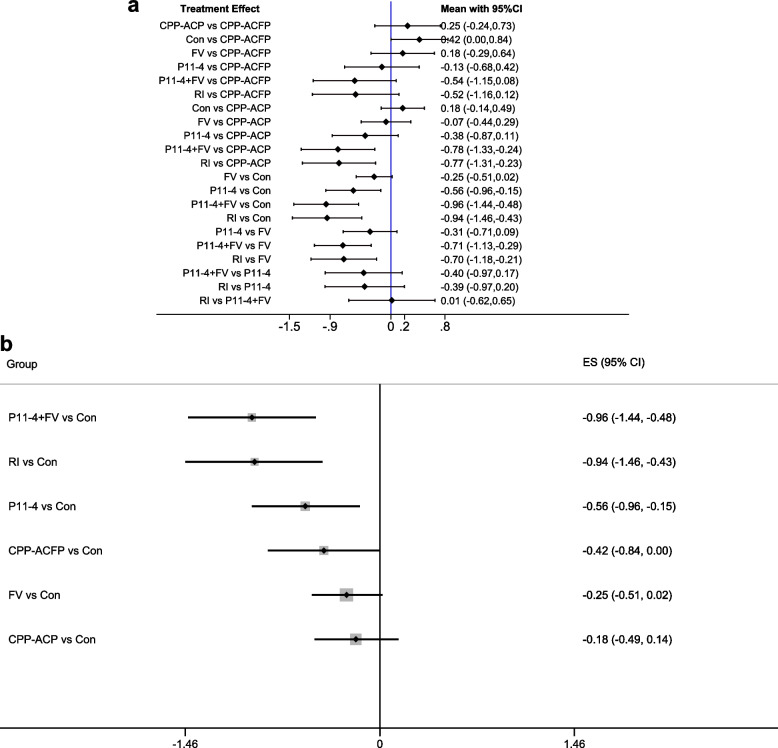
a Forest Plot, b Forest plots for comparison with the control group

Figure 4a shows the NMA forest plot from the consistency model. We used SMD as the effect size. As shown in Fig. 4a, there was a statistically significant difference between 4 groups (P11-4, P11-4 + FV, RI, CPP-ACFP) and the ‘Control’ group (with 95% CI of SMD < 0). Compared to the ‘FV’ and ‘CPP-ACP’ groups, the ‘P11-4 + FV” and ‘RI” groups showed a significant difference (with 95% CI of SMD < 0). No significant differences were found for other comparisons. Visual displays of point estimates and confidence intervals of relative effects of interventions against a common comparator were shown in Fig. 4b [96]. There were no statistically significant differences in direct and indirect comparisons between these interventions and the control group according to inconsistency analysis.

#### SUCRA ranking

**Fig. 5:**
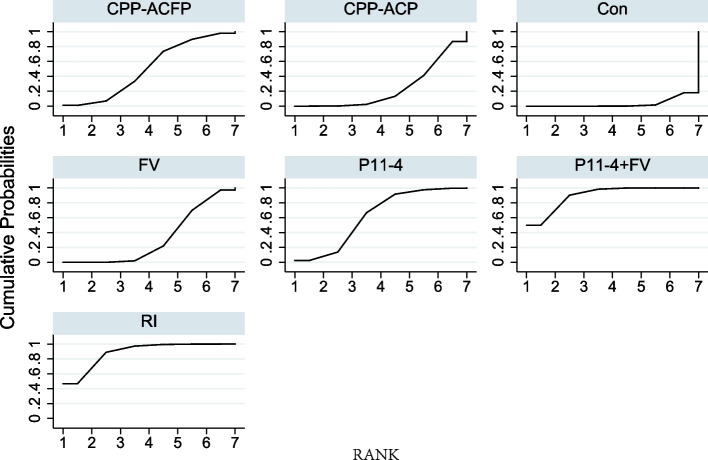
Surface probabilities under cumulative ranking (SUCRA) Values of Seven Therapies. The horizontal axis is the rank sequence from 1 to 7. The vertical axis is cumulative probabilities. Intervention is ranked based on SUCRA. The larger the surface area under the curve and the faster the curve rises, the greater the possibility of being the most efficacious treatment. The specific calculated SUCRA values are shown in Table 4. Con: Control group; FV: Fluoride varnish; P11-4: self-assembling peptide 11–4

**Fig. 6:**
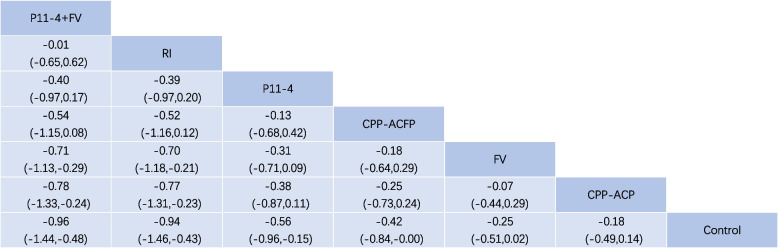
Outcome absolute value changes identified in association with The 7 Therapies

Figure 5 showed the SUCRA of seven therapies. The hierarchy of WSLs' treatments and the SUCRA values are shown in Table 5. The higher the SUCRA value, the higher the ranking. The values of SUCRA used in our study indicated the following hierarchy among the seven treatments: 50.5, 24, 3.3, 31.9, 61.9, 89.7, and 88.7% for the CPP-ACFP, CPP-ACP, Control, FV, P11-4, P11-4 + FV, RI treatments. Figure 6 shows the changes in the absolute value of the outcome identified in association with the seven therapies.

**Table 5:** Hierarchy of seven therapies by SUCRA

Therapies	SUCRA	PrBest	MeanRank
P11-4 + FV	89.7	50.0	1.6
RI	88.7	46.7	1.7
P11-4	61.9	2.4	3.3
CPP-ACFP	50.5	1.0	4.0
FV	31.9	0.0	5.1
CPP-ACP	24.0	0.0	5.6
Control	3.3	0.0	6.8

#### Publication bias

**Fig. 7:**
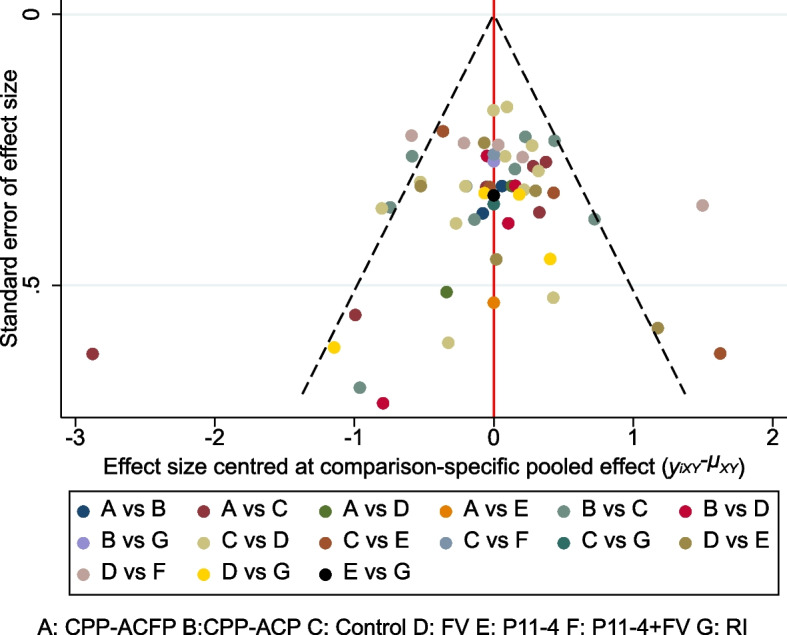
publication bias plot

The funnel plot fitted to the comparison was symmetrical around the zero line, indicating that there was no evidence of publication bias. The publication bias plot is shown in Fig. 7.

## Discussion

We sought to compare the common therapy effects of white spot lesions and searched as much literature as possible for this network meta-analysis. Several valuable findings from this network analysis may inform standardized treatment procedures for the treatment of WSLs. Firstly, the clinical efficacy of conventional fluoride based as well as CPP-ACP-based remineralization strategies is not statistically significant. Secondly, resin infiltration and P11-4-based treatment strategies ranked high. Finally, we have observed that the combination of drugs improves the effectiveness of remineralization therapy in WSLs. In particular, the combination of the self-assembled peptide P11-4 and the fluoride varnish showed the most excellent efficacy.

Based on the SUCRA probabilities, we created an effect size hierarchy for therapeutic effects. The ‘P11-4 + FV’ and ‘resin infiltration’ interventions had more effective outcomes than the other interventions, followed by ‘P11-4’, ‘CPP-ACFP’, ‘FV’, ‘CPP-ACP’, and ‘Control’ interventions. This result suggests that fluorinated varnishes are not clinically effective compared to the control group [ES: -0.25 95%CI (: -0.51,0.02)], even though fluoride strategies are currently the gold standard for managing WSLs [2, 16, 17]. There have been reports of deficiencies in current fluoride therapies, primarily ineffective in caries that have already manifested as white spots [6, 12, 19, 20]. It has already been supposed that the effects restricted to the enamel surface layer led to the shortcomings of fluoride-based strategies [6, 97].

The NMA on the efficacy of CPP-ACP is also under the current clinical status [35, 98–100], with no significant differences either compared to FV [ES: 0.07 95%CI (: -0.29,0.44)] or to the control group [ES: -0.18 95%CI (: -49,0.14)]. CPP-ACP allows for the remineralization of deep lesions [101, 102]. The similarity of CPP-ACP to the fluoride strategy suggests that there are other potential reasons for the remineralization effect. Besides, the study found that SAP P11-4, which can form scaffolds on the enamel surface [6, 27–29, 103], exhibited superior remineralization properties than the control group [ES: -0.56 95%CI (: -0.96, -0.15)]. The effectiveness of P11-4 in randomized studies, conventional Meta-analysis, and the NWA suggest to us that it is more relevant to establish micro scaffolds suitable for remineralization than to provide the required ions for remineralization [12, 15, 30, 31, 69, 103–105].

We need to be more cautious about the effects of resin infiltration therapy, even though it ranks very highly in this analysis [ES: -0.94 95%CI (: -1.46, -0.43) compared to the control group]. Unlike remineralization therapy, resin infiltration, as a minimally invasive etch-adhesive system, can penetrate deep into caries and significantly improve the aesthetic effect of the surface of caries [50, 106, 107]. This means that resin infiltration therapy did not cause regeneration of the enamel, although the effectiveness of resin infiltration has been favored by many clinical studies and meta-analyses [37, 40, 50, 108]. Visual indicators such as visual analog scale (VAS) may introduce a potential bias, which also questions the accuracy of optical indicators [50, 58]. Again, this is the reason we did not include these outcomes in the current study. From the results of this research, CPP-ACFP tended over CPP-ACP, and P11-4 + FV combinations also tended over P11-4 alone. Combination therapy appears to be more appropriate for the treatment of WSLs. The combined application of P11-4 and fluoride varnish holds the highest ranking [ES: -0.96 95%CI (: -1.44, -0.48) compared to the control group], probably due to the formation of precursor scaffolds while providing the ion pool required for remineralization. In summary, the precursor scaffolds and remineralization ion pools together facilitate the management and treatment of WSLs.

We would like to stress here the importance of this study and some methodological necessities. Firstly, there is still no network meta-analysis of WSLs, and in particular, there is a lack of a comprehensive evaluation system for multiple remineralization therapies and resin infiltration therapies. Secondly, there is an urgent need for standardization of current clinical strategies regarding WSLs. Our study will provide an important reference for this. In addition, to match the standardization in the definition of WSLs, we chose 2007 as the starting year for the search. The ICDAS II standards were theoretically discussed in 2005 by the ICDAS work-shop [4, 109, 110]. It’s necessary promoting the changes in caries-related clinical decision-making strategies [111]. It often takes time. It was at the 54^th^ ORCA Congress in 2007 that the ICDAS II criteria became a keyword in the diagnostic section compared to the ICDAS criteria in the 53^rd^ ORCA Congress [112, 113]. Finally, the use of SUCRA alone for comparison of treatment outcomes in NMA is not adequate. Therefore, we used an inconsistency test (Table 4), SUCRA statistic (Table 5 and Fig. 5), and visual displays of point estimates and confidence intervals of relative effects of interventions against a common comparator (Fig. 4b) in this NMA to aid in interpretation [96]. 

We have equally carefully considered the limitations of this study. Most notably, there remains a paucity of trials in this space that can inform direct comparisons, in particular, the top-ranked interventions. The vast majority of direct comparison studies are relative to FV or control groups. Besides, we did not discuss potential influencing factors for WSL, such as gender, age, follow-up time, outcome measuring tool, etc. This is because the data indicating these contents are difficult to unify. Finally, we also recognize the potential bias that comes from setting language limits. However, there was no regional selection bias in this study. We also compared other systematic reviews that were not included in other languages to identify possible bias [114, 115].

Overall, this systematic review and network meta-analysis points to the clinical advantages of resin infiltration and SAP P11-4 (in combination with fluorinated varnish or as a single agent). This study clarifies the hierarchy of multiple therapies for WSLs and informs clinical strategies for WSLs. We plan to attempt analyses of confounding factors in the future to provide more reference value for the standardization of WSLs treatment.

## Conclusions

Our study compared and evaluated the effects of the treatment for WSLs. Both resin infiltration and SAP P11-4 have a positive therapeutic effect on WSLs. The clinical efficacy of both CPP-ACP-based and fluoride-based drugs is not significant. The combination of SAP P11-4 and fluoride varnish is a better strategy for treating WSLs.

## Supplementary Information

**Additional file 1.**

## Data Availability

All data generated or analysed during this study are included in this published article [and its supplementary information files].
