# Supplementary material for: Comparison of therapies of white spot lesions: a systematic review and network meta-analysis
Source: BMC Oral Health. 2023 Jun 1;23:346. doi: 10.1186/s12903-023-03076-x (PMC10233982; doi:10.1186/s12903-023-03076-x)
Supplement: Supplementary file 1 — Additional file 1. [file 12903_2023_3076_MOESM1_ESM.docx]

Supplementary Table Search Strategy of Resources

| Resources | Search Strategy | Research Number |
| --- | --- | --- |
| Pubmed | (("orthodontic brackets/adverse effects"[MeSH Major Topic] OR "dental caries/drug therapy"[MeSH Major Topic] OR "white spot lesion"[All Fields] OR "early caries lesion"[All Fields] OR " non-cavitated lesions "[All Fields] OR "Tooth Remineralization"[MeSH Terms] OR "dental caries/prevention and control"[MeSH Major Topic]) AND ("fluorides/therapeutic use"[MeSH Major Topic] OR "caseins/therapeutic use"[MeSH Major Topic] OR "composite resins/therapeutic use"[MeSH Major Topic] OR "CPP-ACP"[All Fields] OR "Casein Phosphopeptide-Amorphous Calcium Phosphate"[All Fields] OR "Fluoride"[All Fields] OR "Resin Infiltration"[All Fields] OR "Pearl powder"[All Fields] OR "Duraphat"[All Fields]) AND ("fluorides/therapeutic use"[MeSH Major Topic] OR "caseins/therapeutic use"[MeSH Major Topic] OR "composite resins/therapeutic use"[MeSH Major Topic] OR "CPP-ACP"[All Fields] OR "Casein Phosphopeptide-Amorphous Calcium Phosphate"[All Fields] OR "Fluoride"[All Fields] OR "Resin Infiltration"[All Fields] OR "Pearl powder"[All Fields] OR "Duraphat"[All Fields] OR "Placebo"[All Fields] OR "Gum"[All Fields])) AND (randomizedcontrolledtrial[Filter]) AND (“ICDAS” OR “QLF” OR “LF” OR “quantitative light induced fluorescence” OR “laser fluorescence”) | 186  (82 RCTs) |
| Cochrane | Search strategy of Cochrane library is based on the following link: https://www.cochranelibrary.com/advanced-search/search-manager?search=6998211 | 193  (174 RCTs) |
| Embase | ('orthodontic bracket'/exp OR 'dental caries'/exp OR 'dental restoration'/exp OR 'white spot lesion' OR 'early caries lesion' OR 'non-cavitated lesions') AND ('fluoride'/exp OR 'casein'/exp OR 'resin'/exp OR 'cpp acp' OR 'cpp-acfp' OR 'casein phosphopeptide-amorphous calcium phosphate' OR fluoride OR 'resin infiltration' OR 'pearl powder' OR 'fluoride varnish'/exp OR duraphat OR 'self-assembling peptide') AND ('fluoride'/exp OR 'casein'/exp OR 'resin'/exp OR 'cpp acp' OR 'cpp-acfp' OR 'casein phosphopeptide-amorphous calcium phosphate' OR fluoride OR 'resin infiltration' OR 'pearl powder' OR 'fluoride varnish'/exp OR duraphat OR 'self-assembling peptide' OR 'placebo'/exp OR 'control'/exp) AND (qlf OR 'quantitative photoinduced fluorescence' OR 'laser fluorescence' OR lf OR area) | 1946  (236 RCTs) |
| Web of Science | (((((TS=(white spot lesion)) OR TS=(WSL)) OR TS=(early caries lesion)) OR TS=(ECL)) OR TS=(non-cavitated lesions)) OR TS=(Tooth Remineralization) AND  (((((((((((TS=(fluoride)) OR TS=(casein)) OR TS=(resin)) OR TS=(cpp acp)) OR TS=(cpp-acfp)) OR TS=(casein phosphopeptide-amorphous calcium phosphate)) OR TS=(fluoride)) OR TS=(resin infiltration)) OR TS=(pearl powder)) OR TS=(fluoride varnish)) OR TS=(duraphat)) OR TS=(self-assembling peptide) AND (((((((((((((TS=(fluoride)) OR TS=(casein)) OR TS=(resin)) OR TS=(cpp acp)) OR TS=(cpp-acfp)) OR TS=(casein phosphopeptide-amorphous calcium phosphate)) OR TS=(fluoride)) OR TS=(resin infiltration)) OR TS=(pearl powder)) OR TS=(fluoride varnish)) OR TS=(duraphat)) OR TS=(self-assembling peptide)) OR TS=(placebo)) OR TS=(control) AND ((((TS=(qlf)) OR TS=(quantitative photoinduced fluorescence)) OR TS=(laser fluorescence)) OR TS=(lf)) OR TS=(area) | 707  (131 RCTs) |
| Other Meta-analysis | Carefully searched in traditional meta-analysis | 10 RCTs |
